# A Feasibility Study of Using an In-Ear EEG System for a Quantitative Assessment of Stress and Mental Workload

**DOI:** 10.3390/s26020442

**Published:** 2026-01-09

**Authors:** Zhibo Fu, Kam Pang So, Xiaoli Wu, Arthit Khotsaenlee, Savio W. H. Wong, Chung Tin, Rosa H. M. Chan

**Affiliations:** 1 Department of Biomedical Engineering, City University of Hong Kong, Hong Kong; zhibofu2-c@my.cityu.edu.hk (Z.F.); chungtin@cityu.edu.hk (C.T.); 2 MindAmp Limited, Hong Kong; 3 Department of Educational Psychology, The Chinese University of Hong Kong, Hong Kong; savio@cuhk.edu.hk; 4 Department of Electrical Engineering, City University of Hong Kong, Hong Kong

**Keywords:** EEG, wearable sensors, physiological monitoring

## Abstract

While electroencephalography (EEG) is effective for assessing stress and mental workload, its widespread adoption is currently hindered by the complex setup of most existing EEG systems. This article presents a new in-ear EEG system and investigates its feasibility for developing robust models to quantify stress and mental workload levels. The system consists of a single-channel EEG acquisition device that has a similar form factor as user-generic earpieces. All electrodes including passive, reference and bias electrodes were put on the ear, which optimized the device’s usability. We validated the system through two experiments with 66 subjects to collect EEG data under varying stress and mental workload conditions. We developed classification and regression models to predict stress and mental workload levels from the data. Cross-subject stress classification achieved 77% accuracy, while within-subject stress regression yielded an average R2 of 0.76 ± 0.20. Two-class mental workload level classification reached accuracies between 70% and 80% for the arithmetic and finger tapping tasks. Feature importance analysis revealed that frequency-domain EEG features, particularly in the alpha and beta bands, significantly contributed to the models’ performance. However, we observed lower within-subject feature variation and model accuracy for the mental rotation, potentially due to the distance between brain regions engaged and the device’s recording site. Our findings demonstrate the potential of using the presented EEG device to monitor stress and mental workload in real-time.

## 1. Introduction

Electroencephalography (EEG)-based brain–computer interfaces (BCIs) have demonstrated clinical efficacy in medical diagnostics and rehabilitation [1]. Recent advancements in analog front-end design, electrode materials, and digital signal processing have enabled miniaturized wearable EEG devices, expanding applications beyond clinical applications [2,3]. Learning effectiveness depends on appropriate levels of stress and mental workload, and modern personalized education requires tools to monitor these states in real time at the individual level. Learning is a complex process that relies on a delicate balance of cognition and emotion [4]. The levels of mental workload and stress have a direct impact on the effectiveness of one’s learning process [5,6]. Empirical evidence indicates that a moderate level of stress and mental engagement can enhance one’s learning performance, whereas both under- and over-load can impede the acquisition and retention of knowledge [7,8,9]. With the growing emphasis on self-directed and personalized learning in modern educational paradigms, there is a pressing need to monitor and regulate these mental states at the individual level [10,11].

Recent advancements in evaluating mental workload and stress using EEG have been significantly enhanced by deep learning architectures. By combining convolutional neural networks (CNNs), bidirectional long short-term memory (Bi-LSTM), and gated recurrent units (GRUs), one study achieved 97.60% accuracy in stress-level classification on multi-channel EEG recordings from 48 subjects [12]. Another work reported 97.86% accuracy using an attention-based CNN–LSTM model on multimodal signals (ECG and pulse rate) from 34 subjects [13]. For mental workload assessment, an attention-based LSTM model achieved 87.1% classification accuracy [14]. These algorithms show promising results and substantially improve the usability of EEG for mental workload and stress assessment.

Despite these promising algorithmic results, many existing systems rely on multi-channel, scalp-mounted EEG devices that limit everyday usability. Commonly used research-grade systems include the 14-channel Emotiv EPOC headset (used to collect the STEW dataset [15]), Neuroelectrics Enobio [16], and OpenBCI-based configurations [17], all of which require multiple electrodes placed on the scalp. Even single-channel EEG devices such as the NeuroSky MindWave [18], while simpler to set up, still have an appearance and wearing style that are not ideal for daily use. Beyond EEG, recent reviews show that workload and stress can also be estimated using other physiological signals, including heart rate variability, electrodermal activity, respiration, pupil diameter, and other peripheral measures, highlighting the potential of less obtrusive sensing approaches. A recent survey on physiological sensor technologies in workload estimation emphasizes the growing importance of multimodal and wearable sensing strategies in this context [19]. In this work, we focus specifically on EEG-based assessment, while recognizing that integrating EEG with peripheral physiological signals is an important direction for future research.

Since the introduction of ear-EEG in 2011 [20], research has progressively demonstrated its clinical utility beyond experimental settings. Some applications focused on sleep monitoring and mental state monitoring [21,22], investigations have also expanded into neurodegenerative disease screening [23]. Notably, multi-night in-ear EEG recordings have identified distinctive sleep-related EEG biomarkers associated with mild cognitive impairment (MCI) [24]. It has several advantages over other types of wearable EEG devices such as tighter contact between electrodes and skin, and a more stable electrode positioning [25], making ear-EEG a promising tool for long-term EEG monitoring. Furthermore, ear-EEG significantly reduces the setup time and complexity compared to the traditional scalp EEG measurements. Even users without prior experience with EEG devices can easily operate the system by placing electrodes into ears, akin to wearing common earphones. Thus, combining ear-EEG with earphone form factors can greatly increase the usability and user-friendliness of EEG systems in real-life scenarios.

In summary, prior studies have demonstrated that deep learning models applied to multi-channel EEG can achieve high accuracy for mental workload and stress classification, and that various physiological modalities can also be used for this purpose. Nevertheless, most existing systems rely on bulky, scalp-mounted EEG hardware and are evaluated primarily in controlled laboratory settings, which limits their suitability for unobtrusive, everyday use. Furthermore, the potential of compact, wearable EEG form factors for reliable workload and stress estimation remains underexplored. To address this gap, this study proposes a low-cost, scalable in-ear EEG system that adopts the form factor of consumer-grade earpieces and integrates off-the-shelf components. Unlike conventional EEG systems that follow the 10–20 layout, all electrodes in our design are placed on the ear and within the ear canal, greatly improving the portability and usability of the system. We then investigate its feasibility for predicting stress and mental workload levels during learning tasks. By leveraging recent advances in portable EEG technology and machine learning, this work aims to lay the groundwork for adaptive, closed-loop feedback systems that optimize learning outcomes by tailoring instructional interventions to individual learners’ needs. Overall, the proposed approach seeks to balance the ecological validity of real-world educational settings with the rigor of controlled laboratory experiments, thereby helping to bridge the gap between the two.

## 2. Materials and Methods

### 2.1. In-Ear EEG System Design

**Figure 1:**
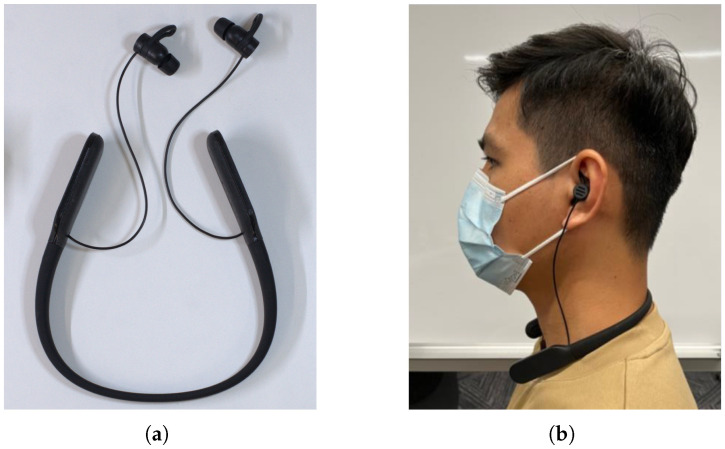

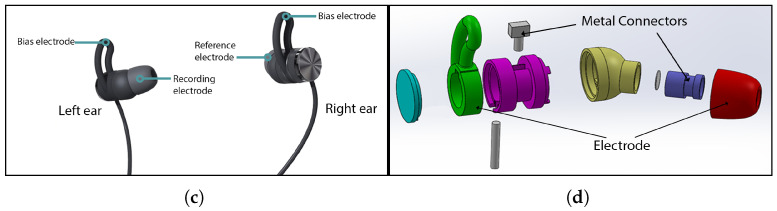
In-ear EEG device design. (**a**) Picture of the fully assembled device. (**b**) Subject wearing the device. (**c**) Electrodes’ locations. (**d**) Structure of the earpiece; the electrodes were put on the metal connectors which were connected to the PCB with wires.

The in-ear EEG system used in this study was designed to resemble a neckband earphone, aiming to enhance user-friendliness and mass manufacturability, as illustrated in Figure 1a. How the device was worn is depicted in Figure 1b. The single-channel EEG acquisition system comprised an ADS1299 analog front-end (Texas Instruments Incorporated, Dallas, TX, USA) operating at a 500 Hz sampling rate. The digitalized signals were transmitted through a Bluetooth Low Energy (BLE) protocol using an FR5086 system-on-chip (Shanghai Frequen Microelectronics Co., Ltd., Shanghai, China). The device was powered by a 240 mAh battery that can last longer than a 4 h consecutive recording. The Printed Circuit Board (PCB), control buttons and USB-C charging port were located on the right side of the neckband, while the battery was housed on the left. The two sides were connected by electric wires running internally within the neckband. To minimize potential interference, the wire for EEG signal transmission that is placed inside the neckband was shielded from the power supply wires running alongside it. The dry electrodes were manufactured by Dongguan Reebelk Silicone Products Co., Ltd. (Dongguan, China); all electrodes were manufactured with silicone material and graphite to achieve both high conductivity and high softness for user comfort. As shown in Figure 1c, two bias electrodes were positioned on the concha site in each ear, with the passive recording electrode in the left ear canal and the reference electrode in the right ear canal. The bias electrodes are shaped like the ear support found in sports headphones to provide the earpiece with a tighter fit and help to decrease motion artifacts, The electrodes were connected to the ADC through metal connectors on the earpiece; the structure is illustrated in Figure 1d.

The recording and reference electrodes are connected to the differential analog negative input 1 (IN1N) and differential analog positive input 1 (IN1P) on the ADS1299; the bias electrodes are connected to the BIAS pins. The ADS1299 is subsequently connected to the BLE chip via the SPI protocol. The in-ear EEG acquisition system presented in this study was also validated and utilized in another study by Fang et al. [22].

### 2.2. Experiment

The experimental protocol received approval from the Survey and Behavioral Research Ethics Committee at the Chinese University of Hong Kong (SBRE-21-0832). All subjects provided informed consent prior to participating in the study. A total of 66 subjects participated both stress and mental workload experiments.

#### 2.2.1. The Stress Experiment

**Figure 2:**
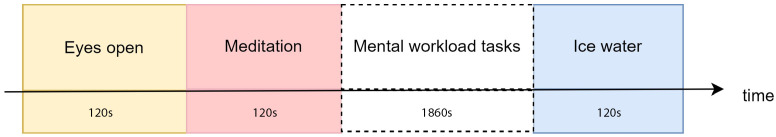
Stress experiment timeline.

The stress experiment aimed to record EEG signals while subjects experienced distinct stress levels. Initially, 51 subjects completed two stress-related conditions: an eyes-open resting task at the beginning of the experiment, representing a relatively lower stress level, and a cold pressor task, which induced a higher stress level. Subsequently, an additional 15 subjects were recruited, for whom an eyes-closed meditation task was inserted after the eyes-open resting task and before the mental workload tasks, in order to induce the lowest stress level among all conditions. First, subjects were asked to perform a training session to familiarize themselves with the task, equipment and surroundings; the training session also helped to reduce the level of nervousness during the following eyes-open resting sessions. Then, they were asked to keep their eyes opened while staying still for two minutes to establish a baseline stress level. At last, subjects were asked to immerse one of their hands in ice-cold water for two minutes to induce a high-stress condition; this cold pressor task was also used in other studies to induce stress responses in the EEG signal [26,27]. The water temperature was kept at 5 °C throughout the immersion. No task randomization was performed for the stress experiment. The cold pressor task aims to induce stress. Putting it at the end of the whole experiment will add to the stress the subject already experienced with a sequence of challenging mental workload tasks and thus represent the highest stress during the whole experiment. Another set of experiments with 15 subjects was added in which the subjects were asked to perform a two-minute closed-eye meditation exercise after resting, a paced breathing exercise where the subjects were guided to control their respiration rate to be around 10 breaths/min, while maintaining a comfortable posture to engage in a low-stress state [28]. Because increased alpha activity can represent lower stress levels [29], this meditation task was included to emulate the lowest stress condition and for exploring the difference of stress level among three different tasks. Following the completion of each two-minute task, subjects were asked to provide a subjective stress rating on a scale from 1 to 9. This self-reported measure served as a valuable source of feedback, allowing for a more comprehensive understanding of the individual’s subjective experience of stress in relation to the objective physiological measures captured by the EEG recordings. The timeline of the study is shown in Figure 2.

#### 2.2.2. The Mental Workload Experiment

**Figure 3:**
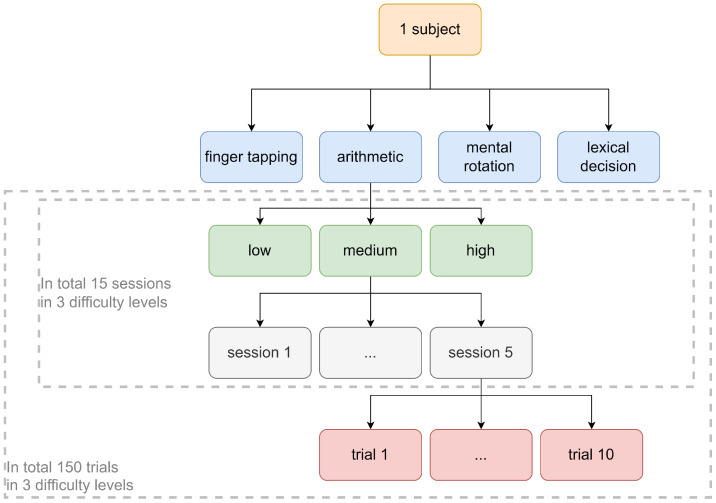
Dataset hierarchy.

**Figure 4:**
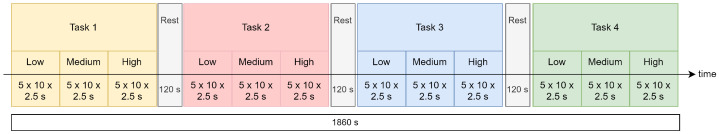
Mental workload experiment timeline.

In the present study, we adopted the same cognitive tasks and mental workload manipulations previously employed by So et al. [11] to investigate the feasibility of using a single-channel, in-ear EEG device for real-time monitoring of mental workload during learning activities. The four cognitive tasks employed in this study were selected to span distinct cognitive and motor domains, and each exhibits robust parametric scalability across difficulty levels, which means the task difficulty can be systematically manipulated along theoretically meaningful dimensions, enabling fine-grained assessment of mental workload variation. This multi-domain approach ensures comprehensive assessment of mental workload across fundamental cognitive processes relevant to learning and problem-solving. The arithmetic task engages numerical reasoning and computational problem-solving, activating the central executive component of working memory alongside higher-order executive functions such as attention allocation and response inhibition. Moreover, these tasks reflect fundamental cognitive and motor functions involved in everyday learning and problem-solving. For example, arithmetic and mental rotation are core components of learning in STEM education, involving numerical reasoning and spatial visualization skills. Finger tapping relates to motor skill acquisition and coordination, which are essential in many learning contexts, including musical training. Lexical decision taps into language processing, vital for reading and verbal learning. In the arithmetic task, subjects were presented with arithmetic equations on a screen and required to determine their correctness. They used the ’left’ and ’right’ arrow keys on the keyboard to indicate their answers. The mental workload levels in this task were manipulated based on the complexity of the calculations involved. The content of the task for each mental workload level was as follows. Low mental workload: Single-digit addition. Medium mental workload: Double-digit addition with carry or subtraction with borrow. High mental workload: Mixed arithmetic operations. For the finger tapping task, subjects engaged in visualmotor coordination. They were presented with a pattern on the screen and required to tap the corresponding keys (‘A’, ‘S’, ‘D’, ‘F’, ‘J’, ‘K’, ‘L’, and ‘;’) on the keyboard. The content of the task varied across different mental workload levels, as follows. Low mental workload: Involves a single hand and a single finger. Medium mental workload: Involves a single hand and multiple fingers. High mental workload: Involves two hands and multiple fingers. In the mental rotation task, subjects were presented with pairs of polyomino/polycube figures and tasked with determining whether they represented the same figure or not. The polyomino/polycube figures could either be distinct figures or the same figure viewed from different perspectives. These polyomino/polycube figures were composed of interconnected small squares/cubes. Specifically, polyomino figures that consisted of 2D squares were used in the low mental workload condition. In contrast, polycubes constructed using 3D cubes were used in the medium mental workload condition, whereas polycubes with the highest number of cubes were used in the high mental workload condition. In the lexical decision task, linguistic ability was involved. Subjects were presented with words on the screen and required to determine if the word was a real English word or a pseudo-word. The words at different mental workload levels were created by varying factors, such as the word usage, number of characters, and part of speech. The pseudo-words in this task were generated using Wuggy software v1.0.0 [30]. Within each task, there were three levels of mental workloads: low, medium, and high. To ensure a robust and reliable assessment of each mental workload, each level within a task consisted of five separate sessions, with each session comprising 10 trials. This hierarchical structure allowed for a fine-grained analysis of the EEG data, capturing both within-subject and between-subject variability in neural responses to mental workload. The hierarchical relationship within the dataset is described in Figure 3. The time given for each trial was 2.5 s. The time given for resting was 120 s. The maximum total time required for the experiment was 1860 s. The timeline of the whole study is shown in Figure 4.

### 2.3. Data Collection

Prior to analysis, the collected data were subjected to a rigorous quality control procedure to minimize potential confounds and ensure the integrity of the results. At the subject level, data files were carefully screened based on two key criteria: signal quality and file integrity. Immediately following each subject’s experimental session, the signal quality was evaluated using a five-point scale, with 1 representing the poorest quality and 5 indicating the highest quality. This rating was assigned based on a meticulous observation of the subject’s behavior during the experiment, with particular attention paid to factors that could introduce artifacts into the EEG recording. For example, subjects who exhibited excessive movement, resulting in discernible motion artifacts, received lower scores on the quality scale. To ensure that only data of sufficient quality was included in the analysis, a signal quality threshold was established, and any subject data associated with corrupted files was excluded from further consideration. At the segment level, an additional layer of quality control was implemented to identify and retain only those signal segments that met predetermined voltage and flat signal criteria. The voltage criterion was designed to exclude segments with signal saturation, defined as any segments with a magnitude range larger than 80 μV. The flat signal criterion was designed to exclude segments that did not contain useful information, defined as any segments with a magnitude range less than 1 μV. By applying these stringent criteria, we ensured that the data used in the stress and mental workload studies consisted solely of high-quality, informative segments that could provide reliable insights into the underlying neural processes. The data screening process was the only method used for data selection throughout the study. This approach ensures data integrity and provides a reliable basis for interpreting and generalizing the findings from the stress and mental workload experiments. The stress experiment included a total of 66 subjects. To ensure the integrity and reliability of the data, a rigorous screening process was implemented through data package sequence verification and visual data inspection. Subjects whose data were contaminated with an excessive amount of artifacts were excluded from further analysis. After this comprehensive screening process, the final sample consisted of 55 subjects (17 males, age ranging from 18 to 22), whose EEG recordings were deemed to be of sufficiently high quality for inclusion in the study, including 8 subjects in the meditation. Using the same screening criteria, EEG data of 44 subjects (8 males, age ranging from 18 to 22) were deemed to be of adequate quality for inclusion in the mental workload study. The mental workload task took much longer time than the stress task; some subjects introduced a significant amount of artifacts to the data, such as motion artifacts and muscle artifacts. Those data were marked as invalid during the experiment thus only 44 were selected.

### 2.4. Data Processing

#### 2.4.1. Pre-Processing

**Figure 5:**
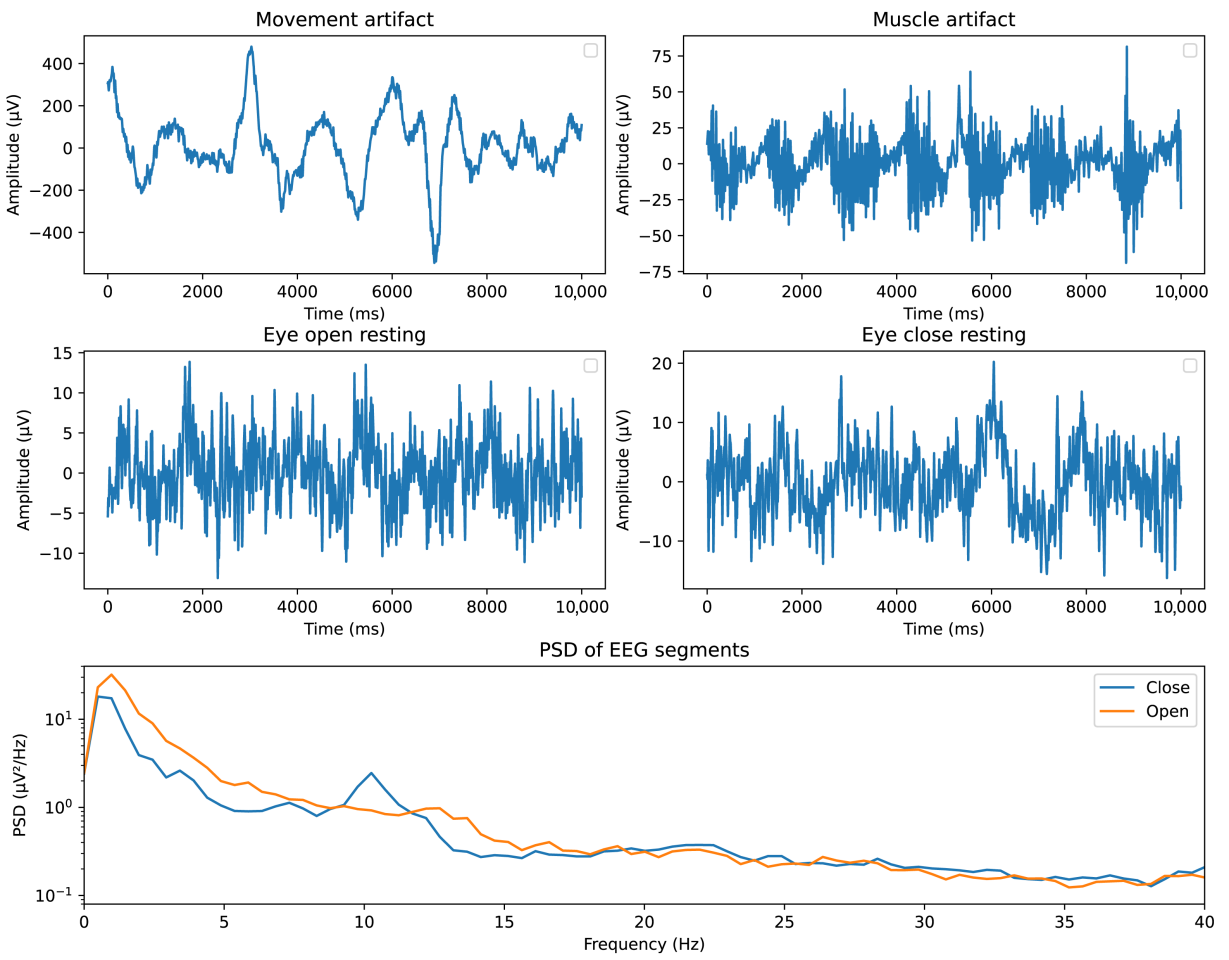
Sample signal from the EEG system from top to bottom: Data without significant artifacts. Data contaminated with large amplitude movement artifacts (head movement). Data contaminated with muscle artifacts (mouth movement, chewing, etc.).

After acquiring the raw EEG data, a series of pre-processing steps were performed to reduce the noise and enhance the efficiency of feature extraction. The pre-processing pipeline began with a preliminary screening to identify and remove files with a high likelihood of containing contaminated data. Subjects with missing EEG files or incorrect data input, such as erroneous time stamps, were excluded from further analysis. This initial step served to ensure the integrity and reliability of the dataset, minimizing the impact of corrupted or incomplete recordings on subsequent processing and analysis. Following the initial screening, a high-pass filter with a 1 Hz cutoff frequency, a low-pass filter with a 49 Hz cutoff frequency, and a 50 Hz notch filter were employed to remove power-line noise and high-frequency muscle artifacts. As the experiments were conducted within a controlled environment, with the experimenter monitoring the signal during the whole experiment, a simple threshold-based method was used for eliminating data chunk contaminated with a large number of artifacts. There are two common types of artifacts in our data: movement artifacts and muscle artifacts. The typical amplitude of the collected EEG data after filter is within ±20 μV; signal chunks exceeding this threshold are considered as contaminated, as shown in Figure 5. To prepare the data for analysis, the pre-processed EEG signals were segmented differently for the stress and mental workload studies. In the stress study, the signals were divided into 3 s segments, providing a temporal resolution suitable for capturing the dynamic changes in stress levels. In contrast, for the mental workload study, each trial was treated as a single segment, preserving the integrity of the task-related neural activity. A critical consideration in the stress study was the imbalance in the number of signal segments obtained from different tasks. This imbalance stemmed from the varying number of subjects participating in each task, with only 429 segments from the meditation task, 1258 segments from the eyes-open task, and 737 segments from the cold pressor. If left unaddressed, this imbalance could introduce significant biases and compromise the validity of classification tasks performed on the original data proportions. To mitigate this issue, a class balancing technique was implemented prior to training the stress classification models. This involved randomly sampling from the larger classes to achieve an equal number of samples in each class. By ensuring a balanced representation of the different stress conditions, the class balancing step aimed to prevent the classifiers from being unduly influenced by the more prevalent classes, thus improving the generalizability and robustness of the models. It is important to note that class balancing was not performed for the regression tasks in the mental workload study. The nature of regression analysis, which aims to capture the continuous relationship between EEG features and mental workload levels, does not require the same balancing considerations as classification tasks, where discrete class labels are assigned.

#### 2.4.2. Time Series Features and Labeling

Global time series features were extracted from the raw EEG signal for the classification process. There are three main types of features utilized: statistical features, Hjorth features and frequency domain features. Each category of features captures distinct aspects of the EEG signal, providing complementary information for characterizing the underlying neural activity. Statistical features, which are not domain-specific, are commonly employed to describe the dispersion and shape of data distributions. These features include standard measures such as standard deviation, mean, skewness, and kurtosis. By quantifying the central tendency, variability, and higher-order moments of the EEG signal, statistical features offer a general assessment of the signal’s properties in the time domain. Hjorth features, on the other hand, are features of a times series bridging the gap between the time domain and the frequency domain. They can be derived from the power spectrum of the time series [31]. The Hjorth features used are Hjorth complexity, mobility and activity. These features provide insights into the signal’s complexity, the rate of change, and the overall power, respectively, capturing both temporal and spectral characteristics of the EEG signal. Frequency domain features are specifically tailored to the analysis of EEG signals. In this study, the EEG signal is decomposed into five distinct frequency bands: delta (0.1–4 Hz), theta (4–8 Hz), alpha (8–12 Hz), beta (12–32 Hz), and gamma (32–45 Hz). These frequency bands are widely recognized in the EEG literature and are associated with various cognitive and neurophysiological processes. By examining the power and other characteristics of the EEG signal within each frequency band, valuable information can be extracted for labeling and further analysis. Additionally, the spectral edge frequency, which represents the frequency below which a specified percentage of the total power is contained, is also considered as a feature in this study. The full list of the above-mentioned features can be found in Appendix A. To generate the ground truth for supervised learning, we assigned labels to the EEG feature vectors based on the experimental conditions and subjective reports. For stress classification, data segments were labeled according to the task being performed. For binary classification, segments recorded during the meditation task were labeled as low stress, while segments from the cold pressor task were labeled as high stress. For stress regression, The target variable for the regression models was the subjective stress rating (scale 1–9) provided by the participant immediately after each task. All EEG feature vectors extracted from a specific task block were assigned the single subjective rating given for that block. For mental workload classification, labels were determined by the predefined difficulty levels of the cognitive tasks.

#### 2.4.3. Model

To address the classification tasks in this study, we evaluated several well-established machine learning models that balance computational efficiency with classification performance. For mental workload classification, we selected support vector machines (SVMs) [11], Random Forest [32,33], artificial neural networks (ANNs) [34,35], and logistic regression. Given the moderate complexity of the task and the constraints of deploying models on resource-limited wearable devices, we deliberately excluded deep learning-based approaches. Logistic regression was included as a baseline model to establish a reference for performance comparison. A preliminary two-class classification was conducted to distinguish between the lowest and highest levels of task difficulty as a proof of concept. Among the tested models, the ANN achieved the highest classification performance. For example, in stress classification task, the ANN achieved an accuracy of 0.78 ± 0.05, while SVMs achieved 0.75 ± 0.06, logistic regression achieved 0.72 ± 0.07 and Random Forest achieved 0.76 ± 0.05. The better performance of the ANN over linear models and tree-based models suggests that stress classification benefits from learning smooth non-linear decision boundaries across the EEG features. The feature space includes interactions between frequency bands (e.g., alpha/beta ratios) that may be better captured by the continuous activation functions in ANNs. Furthermore, the ANN can be further quantized and pruned for deployment on low-power devices.

For the regression task predicting continuous stress levels, we investigated multiple deep learning architectures given the increased complexity compared to binary classification. We used simple linear regression as a baseline and evaluated additional architectures beyond the ANN used in classification, namely convolutional neural networks (CNNs) and long short-term memory (LSTM) networks [36,37,38]. After a comprehensive performance comparison across all models, we found that relatively simple ANNs with four or fewer hidden layers achieved performance comparable to or exceeding that of more complex architectures. Specifically, for the stress regression task, simple linear regression yielded R^2^ < 0.7, while CNN and LSTM models demonstrated only modest improvements with an R^2^ value around 0.8. Given the targeted deployment of these models on wearable devices with constrained computational resources, we selected the simple ANN as the optimal choice for the regression tasks. This decision prioritizes practical applicability over marginal performance gains, as the computational overhead and increased inference latency of complex architectures would compromise the real-time monitoring capability essential for wearable applications.

The models were trained using a cross-entropy loss function for classification tasks and mean squared error (MSE) for regression, with the Adam optimizer applied throughout. ReLU activation functions were used in all hidden layers. For the output layers, sigmoid activation was employed in binary classification, softmax for three-class classification, and a linear activation function for regression. This architecture was chosen for both classification and regression due to its balance between simplicity and effectiveness. The ANN architecture consists of a sequential network that processes input features through a series of linear transformations. The model begins with an input layer of 35 units, followed by three fully connected layers with decreasing dimensions of 20, 10, and 5 units, respectively. Each hidden layer uses ReLU activation, while the output layer consists of a single unit with a sigmoid activation function for binary classification tasks. For the three-class classification problem, the output layer contains three nodes with a softmax activation.

#### 2.4.4. Training

Cross-validation was performed to ensure a fair evaluation of the model using different subsets of the dataset. For classification studies containing multiple subjects, group shuffle split with the subject ID as the group label was used, such that one subject could not have sessions in both the training and the validation set (i.e., a K-subject-out strategy). Otherwise, if the intra-subject variance could be detected by the model in the training phase then it would not be general enough for evaluation when predicting in the validation phase. The shuffling under this circumstance could prevent non-random assignment to the training and validation sets. For studies containing a single subject, a K-fold split on recordings was used. In both the group shuffle split and K-fold split, three-fold splits were used to perform cross-validation.

#### 2.4.5. SHAP Feature Importance

SHAP (SHapley Additive exPlanations) was used in this study to analyze feature importance. SHAP feature selection method makes use of the Shapley value, which can help us to explain the difference between the actual prediction and the average prediction for each prediction instance in the classification tasks [39]. Each feature in the prediction model contributes a certain amount to this difference. The Shapley value is the average marginal contribution to the above difference across all possible combinations of other features. The mathematical expression of the Shapley value of feature i is shown in Equation (1).

(1) $$\phi_{j}\left(val\right)=\sum_{S\subseteq \{1,\ldots ,p\}\setminus j}\frac{|S|!(p-|S|-1)!}{p!}\left(val\left(S\cup \left\{j\right\}\right)-val\left(S\right)\right)$$

where S is a subset of the features used in the model and p is the number of features. val(S) is the prediction for feature values in set S that are marginalized over features that are not included in set S.

## 3. Results

### 3.1. Stress Experiment

#### 3.1.1. The Subjective Stress Ratings vs. Task Conditions

**Figure 6:**
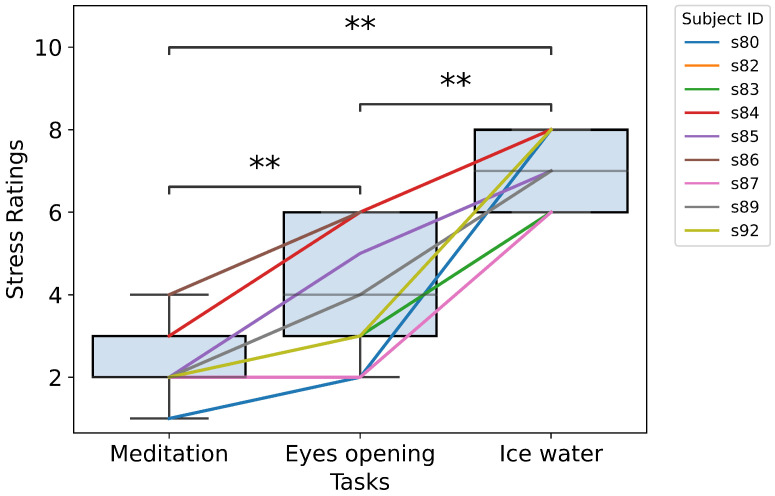
Subjective stress ratings vs. stress tasks. The x-axis shows the stress tasks and the y-axis shows the stress rating of all the experiment trials. Each data point in the box-and-whisker diagram represents one stress rating grouped by the subject ID and task. The significance level of the Mann–Whitney U test was set at 0.05. After the Bonferroni correction, results with *p* < 0.0033 are marked with **. Each line with a unique color represents the stress ratings of one subject across the stress tasks. (Some subjects do not exist in this graph because their data did not pass the quality checks).

Each stress condition in the stress experiment is used to match a certain stress level. The presumed task order with an increasing stress rating is meditation, eyes-open resting and cold pressor. To make sure these conditions are representative enough for the stress levels, a Mann–Whitney U test was performed on the subjective stress ratings of each pair of values. The significance level for the test was set at 0.05. After a Bonferroni correction, results with *p* < 0.017 were marked with *, and results with *p* < 0.0033 were marked with **. From Figure 6, we can observe that the tests on all condition pairs have significant results, indicating different stress levels. When we focus on the line plots for each subject, we can see an increasing trend in stress ratings with the task order. This is further supported by a one-way repeated-measure ANOVA test. The *p*-value of the test is 4.81 × 10^−8^, which is a significant value, indicating a significant difference in stress ratings over tasks. These imply that the stress tasks can induce sequential separable stress levels as presumed. They are suitable for representing the objective stress levels in the stress experiment.

#### 3.1.2. General Classification Model for Objective Stress

In this section, we would like to first mention the classification model performance when incorporating the meditation, eyes-open resting, and cold pressor tasks into the stress study. Table 1 shows the results of the binary classifications of eyes-open resting vs. cold pressor and meditation vs. cold pressor based on EEG features. Within most of the folds, the metrics are steady. The maximum accuracy values of the eyes-open resting vs. cold pressor model and the meditation vs. cold pressor model are 0.84 and 0.92, respectively, for all valid subjects. The average accuracy values of the eyes-open resting vs. cold pressor model and the meditation vs. cold pressor model are 0.78 and 0.77, respectively. For binary classification, the baseline is a random guess with 50% accuracy. A binomial test yielded *p* < 0.001, indicating that our classification performance is significantly better than random guessing. A three-class classification model was also trained to test whether the model can recognize the different stress levels; the classification accuracy was around 0.51, which is below the expected value. This indicates potential challenges in experimental design and subject selection and will be discussed in later sessions.

#### 3.1.3. Subject-Specific Regression Models for Subjective Stress Ratings

**Figure 7:**
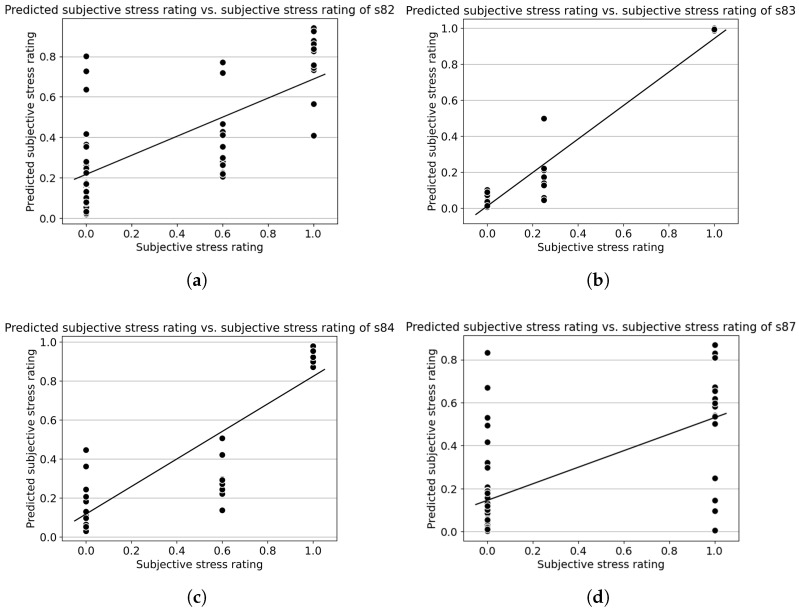
Predicted subjective stress rating vs. subjective stress rating sample regression results. Each data point in the scatter plots represents a pair of subjective stress rating and predicted subjective stress rating. The x-axis shows the subjective stress rating. The y-axis shows the predicted subjective stress rating. The straight line shows the best-fit line of the data. (**a**) Predicted subjective stress rating vs. subjective stress rating of s82. (**b**) Predicted subjective stress rating vs. subjective stress rating of s83. (**c**) Predicted subjective stress rating vs. subjective stress rating of s84. (**d**) Predicted subjective stress rating vs. subjective stress rating of s87.

Table 2 shows the results of the best fold of the regression models of each individual subject. The models predict the subjective stress ratings based on the EEG features. Half of the models can reach an R2 value larger than 0.8 (in bold). Overall, the R2 values are 0.76 ± 0.20. Figure 7 shows some examples of predicted level against subject ratings. A baseline regression model that predicts the mean for all samples yields R^2^ = 0 by definition. Our within-subject models achieved R^2^ = 0.76 ± 0.20, substantially exceeding this null baseline.

#### 3.1.4. Feature Importance

**Figure 8:**
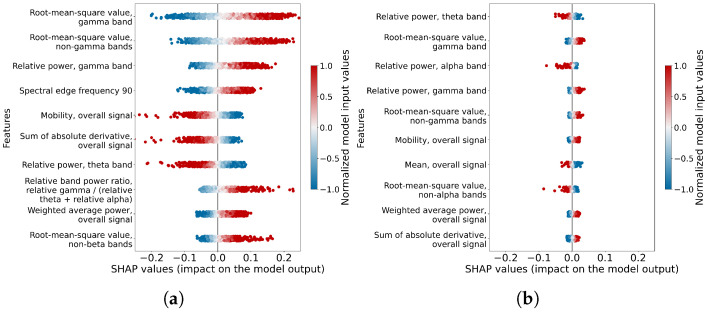
Beeswarm plot of information-dense summary of how the top features in each dataset impact the stress level classification models’ output. (**a**) The important features in the best fold of the stress level classification model for meditation vs. cold pressor. (**b**) The important features in the best fold of the stress level classification model for eyes-open resting vs. cold pressor.

In each mental stress level classification model, the contribution of different features to the final outputs varies, directly influencing the classification results. To quantify these feature contributions and their relative importance, we employed SHAP values. The results of the SHAP analysis were plotted in beeswarm plots (as shown in Figure 8). In the beeswarm plot, each row displays the impact of one feature from all data points (instances). Each dot displays the impact of one feature from one data point. The x position of the dot is determined by the SHAP values of the feature, and the dots ‘pile up’ along each feature row to show density. The color of each dot represents the magnitude of a feature from one data point normalized to the range −1 to 1. The color map diverges from white (representing 0) to red (representing 1) and blue (representing −1). The rows are ordered by the importance of the features. The importance is defined by the mean absolute SHAP values. This definition puts emphasis on the broad average impact of each feature. Only the top ten features are displayed in each subfigure. The SHAP analysis showed that seven features are important for both classification meditation vs. cold pressor and eyes-open resting vs. cold pressor, which are ‘Root-mean-square value, gamma band’, ‘Root-mean-square value, non-gamma bands’, ‘Relative power, gamma band’, ‘Mobility, overall signal’, ‘Sum of absolute derivative, overall signal’, ‘Relative power, theta band’ and ‘Weighted average power, overall signal’. Three out of the seven most important features all relate to the gamma band, which indicates a high importance of gamma band information in stress analysis.

### 3.2. Mental Workload Experiment

#### 3.2.1. Binary Classification Models for Objective Mental Workload

**Figure 9:**
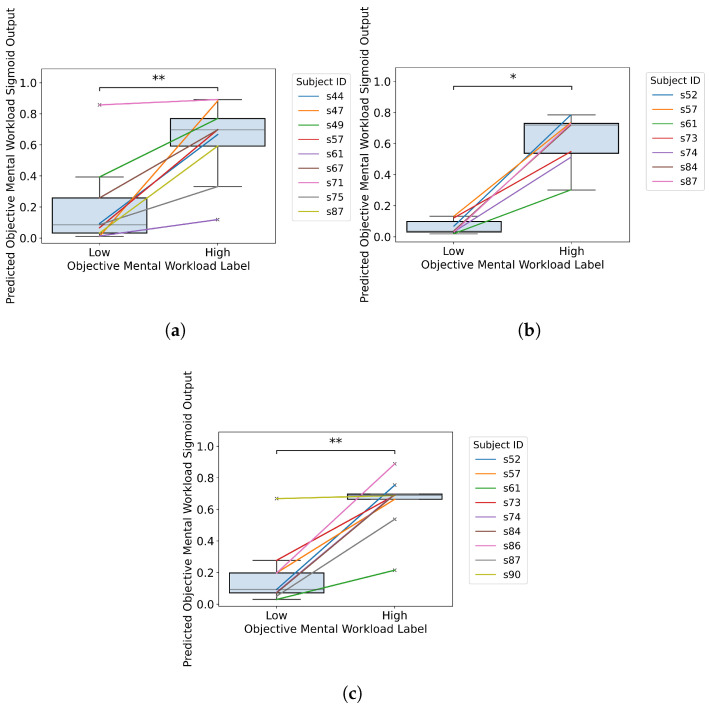
Predicted median sigmoid output of mental workload of the model trained from the arithmetic and finger tapping tasks. Each data point in the box-and-whisker diagram represents one median sigmoid output from one session grouped by subject IDs and task mental workload levels from the objective mental workload classification model. The x-axis shows the objective mental workload level. The y-axis shows the predicted objective mental workload output of the validation trials from the classification model. The significance annotations indicate the significance of the Mann–Whitney U test between the two mental workload levels (* *p* ≤ 0.05, ** *p*≤ 0.01). Each line with a unique color represents the median of the predicted workload. (**a**) Predicted sigmoid output of the mental workload of the model trained from the arithmetic task. (**b**) Predicted sigmoid output of the mental workload of the model trained from the finger tapping task. (**c**) Predicted sigmoid output of the mental workload of the model trained from the arithmetic and finger tapping task together.

The binary classification models were trained with the EEG signal features from four mental workload tasks and their combination. EEG features were input for the model to determine if the task mental workload was low or high. From Table 3, out of the single mental workload tasks, models of arithmetic and finger tapping generate average accuracy values higher than 0.7. This is comparable to [11], in which the accuracy values of models of arithmetic, finger tapping and lexical decision were greater than 0.7, while the accuracy of the model of mental rotation had a lower accuracy of 0.64. To explore the generalization ability of features and models, the EEG data from the arithmetic and finger tapping tasks, both generating models having high predictive accuracy values, were combined to train a new binary classification model. Table 3 shows that the average accuracy is still higher than 0.7. This indicates that the highly accurate prediction models can be further generalized for different tasks based on the original set of EEG feature inputs. Binomial tests confirmed that classification accuracies above 70% were statistically significant (*p* < 0.001), demonstrating performance substantially better than random guessing. The median sigmoid outputs of the neural networks of each subject from experiment trials are plotted in Figure 9. Generally, for each subject, the median predicted sigmoid outputs of mental workload show an obvious trend from the low mental workload to the high one. This is further testified by the Mann–Whitney U test results.

#### 3.2.2. Feature Importance

**Figure 10:**
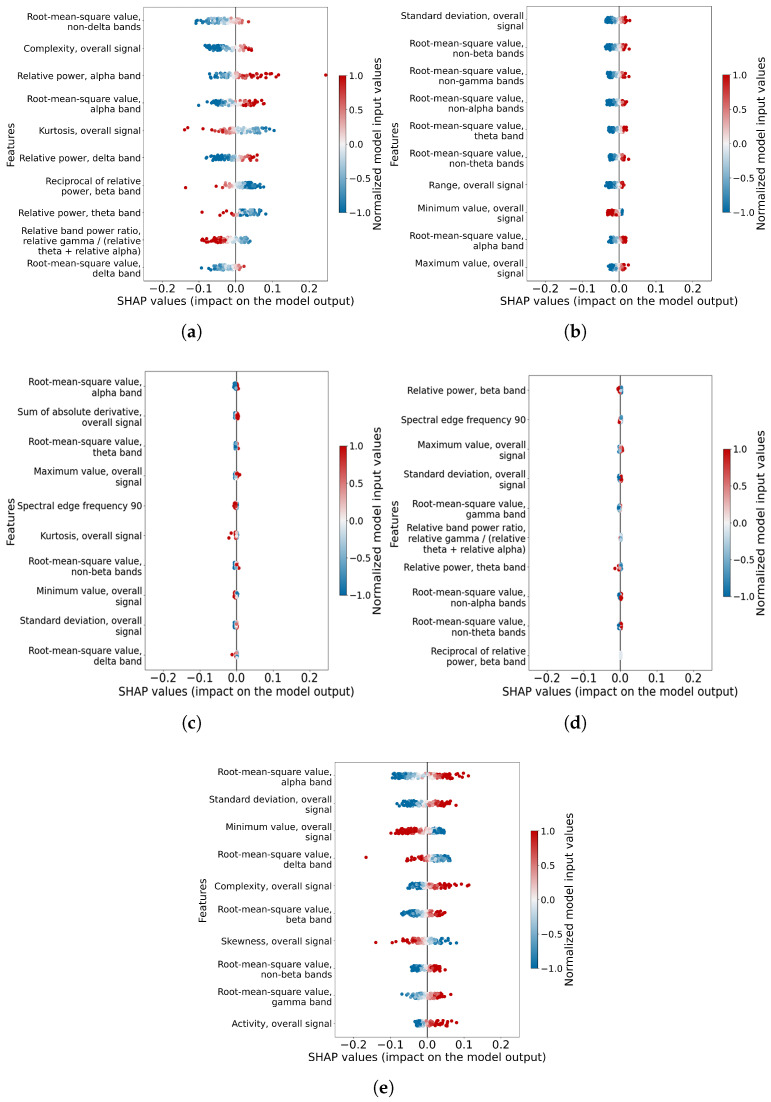
Beeswarm plot of information-dense summary of how the top features in each dataset impact the mental workload classification models’ output. (**a**) The important features in the best fold of the arithmetic mental workload level classification model. (**b**) The important features in the best fold of the finger tapping mental workload level classification model. (**c**) The important features in the best fold of the lexical decision mental workload level classification model. (**d**) The important features in the best fold of the mental rotation mental workload level classification model. (**e**) The important features in the best fold of the arithmetic and finger tapping mental workload level classification model together.

The SHAP values for each feature across different mental workload tasks are visualized using beeswarm plots in Figure 10. From the figure, we can observe that only the EEG features from the arithmetic and finger tapping tasks and their combination have SHAP values that are deviated from zero. This shows the features from these tasks that have contributed to their models. On the other hand, for the EEG features of the mental rotation and lexical decision tasks, the SHAP values are concentrated at zero. This represents the features from these two tasks that are not informative enough for the classification. It can be observed that the datasets with better feature importance can generate better classification models. To better investigate the feature contributions to the models, a subset of important features was extracted and named ‘critical features’. The assumption underlying this analysis is that features with high importance and consistent presence in multiple tasks may possess greater generalizability for mental workload classification. We established a threshold for critical feature selection based on an appearance count of seven or more times in the top ten SHAP value lists across tasks. This procedure yielded five critical features: ‘Complexity, overall signal’, ‘Root-mean-square value, alpha band’, ‘Kurtosis’, and ‘Relative power, beta band’. These features span both the time-domain and frequency-domain characteristics of the EEG information, which are crucial for accurate mental workload classification. Further investigations of the critical features of each task may bring some insights into the different mental workload classification ability of models from different tasks.

#### 3.2.3. Intra-Subject Critical Feature Variation

**Figure 11:**
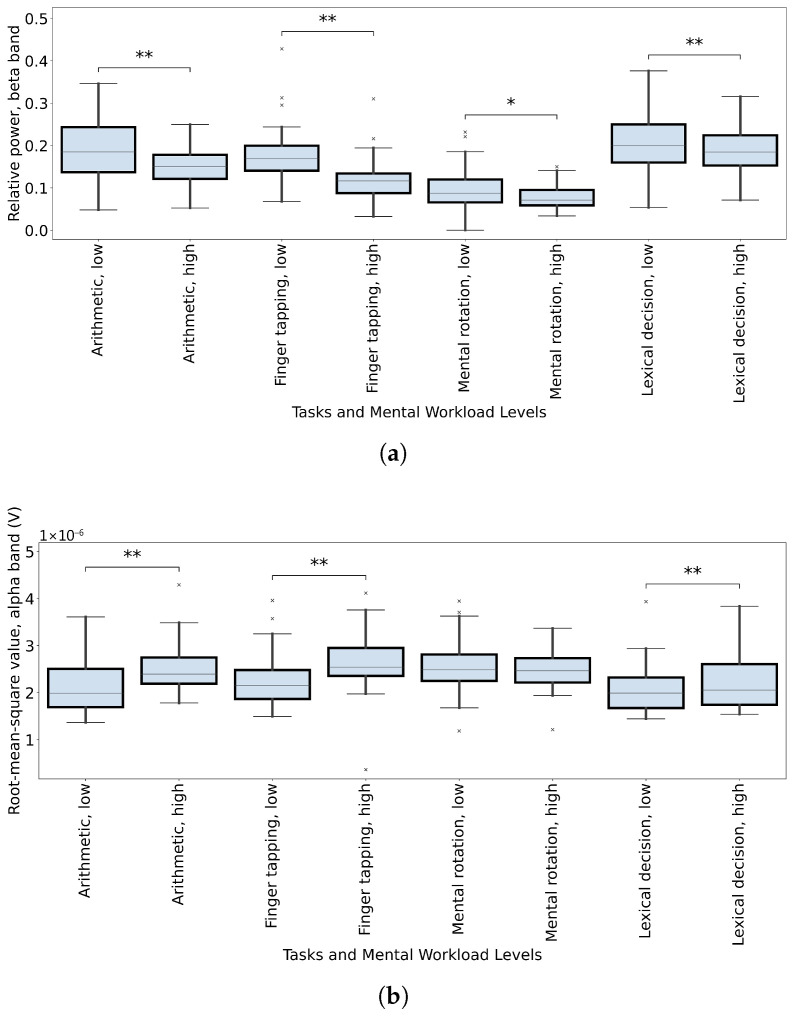
The intra-subject comparison of the mean EEG features within the datasets grouped by the subject ID, mental workload task, and mental workload level. Each data point in the box-and-whisker diagram represents one mean EEG feature value. The x-axis shows the combinations of mental workload tasks and mental workload levels. The y-axis shows the magnitude of the EEG features. A Wilcoxon signed rank test is performed for each pair of feature magnitude of one subject across two mental workload levels, which is the intra-subject variation within each task. The significance annotations indicate the significance of the Wilcoxon signed rank test between the two mental workload levels (* *p* ≤ 0.05, ** *p* ≤ 0.01). (**a**) The intra-subject comparison of the root-mean-square value, alpha band. (**b**) The intra-subject comparison of relative power, beta band.

Data from different mental workload tasks can generate mental workload level prediction models with a range of accuracy values. The above results imply that important features in different tasks can have a significant difference in contributions to the models. Further investigating the critical features may bring some insights into the underlying mechanism. In Figure 11, two frequency-band-related critical features, ‘Root-mean-square Value, Alpha Band’ and ‘Relative power, Beta Band’, are displayed. Each critical feature is presented in a box-and-whisker diagram. Each box contains the mean values of the critical features of one mental workload level of populations of the critical feature from the low mental workload data, and the high mental workload data across subjects are compared with the Wilcoxon signed rank test. This is the investigation of the intra-subject variation of the feature. It is observed that for both frequency-band-related critical features, only arithmetic, finger tapping and lexical decision tasks have very significant levels. The comparison of the mental rotation task is either not significant or just normally significant (*). This finding, highlighting the particular relevance of alpha and beta band features for certain tasks, potentially explains the lower classification accuracy for the mental workload mental rotation tasks. Theta oscillation is traditionally associated with drowsiness and memory consolidation; it shows inconsistent patterns in some workload studies [40,41,42]. Gamma oscillation frequencies overlap with electromyographic (EMG) signals, requiring a high signal-to-noise ratio due to the amplitude typically being smaller than 1 μV [43]. Both theta and gamma bands have some limitations, so alpha and beta bands are selected to analyze the intra-subject critical feature variation.

## 4. Discussion

**Figure 12:**
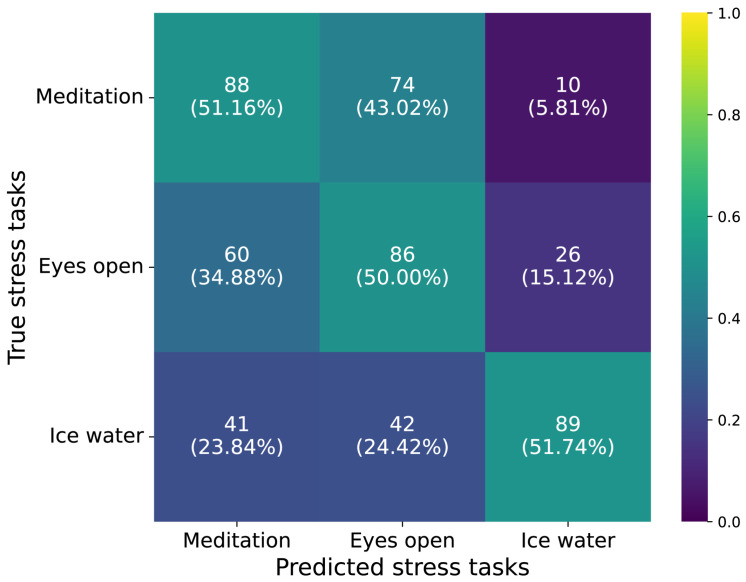
Confusion matrices of the classification result of meditation, eyes-open resting and cold pressor tasks. This matrix provides a detailed view of the model’s performance in predicting stress tasks. The y-axis represents the true labels, while the x-axis represents the predicted labels. Each cell in the matrix corresponds to the number of times a particular true label was predicted as a certain label by the model. The color bar indicates the relative magnitude of the cell values. A darker color represents a smaller value, while a lighter color represents a larger value. Ideally, only the diagonal cells from the top-left to the bottom-right should be filled, indicating that all predictions match the true labels. This would mean that the model’s classification results are fully accurate. Any off-diagonal values represent misclassifications.

Our study examined the feasibility of using a single-channel in-ear EEG device in monitoring stress and mental workload. The result demonstrated that the model could distinguish between high and low stress with an accuracy around 77% for a cross-subject model and an average R2 of 0.76 ± 0.20 for the within-subject model. For mental workload models, it can accurately classify high and low mental workload levels with an accuracy between 70% and 80%. In the stress study, meditation, eyes-open resting and cold pressor task were conducted to induce different stress levels. However, according to the confusion matrix, as shown in Figure 12, a three-class classification model is prone to predicting eyes-open resting as meditation and vice versa. Even though the subjective ratings showed a significance difference between meditation and eyes-open resting, a study [44] showed that novice meditators showed a lower difference in resting and meditation compared with experienced ones. As most subjects recruited in our experiment were non-meditators, the phenomena seen in the confusion matrix is in accord with this finding. Thus, binary classifications between eyes-open resting against cold pressor task and meditation against cold pressor task were conducted instead. They both reached comparable accuracy with another study with single-channel EEG as reported in [45]. Results are also comparable with the results from commercial products with only one channel, which have an accuracy of 0.77 [46].

For stress regression results, a similar study conducted by [47] achieved an R2 value of 0.92 ± 0.02 on a signal from the Fp1, Fp2, F5 and F6 channels. According to [48], stress demonstrated functional brain asymmetry, which is difficult to record with a single-channel EEG setup, and this may lead to an inferior overall regression accuracy compared with multi-channel studies. Some subjects, such as s87 in Figure 7d, may not be accurate due to their self-rating, where the subject rated both eyes-open resting and meditation as the same lowest stress level. Different individuals have various levels of metacognition skill, and this may affect rating abilities [49]. This may explain a low R2 value of 0.41 compared to the average value; low accuracy from subjects like s82 might also be due to this issue. Overall, the system demonstrated its ability to monitor stress levels with good accuracy except for some individuals. Further work is needed to explore the reasons for these outliers and improvement methods. Moreover, While the cold pressor task effectively induced stress, it primarily reflects a physically driven response that may differ from the cognitive or psychosocial stressors relevant to learning contexts. Therefore, the present findings serve as a feasibility demonstration rather than a full validation across mental stress types. Moreover, as gamma-band activity can overlap with pain-related muscle artifacts, the observed features should be interpreted cautiously, and future work should validate the in-ear EEG system under purely cognitive stressors while controlling for potential EMG confounds.

For mental workload tasks, the binary prediction models for arithmetic and finger tapping tasks exhibit higher accuracy compared to those of lexical decision and mental rotation. This discrepancy may be due to the task-triggered signals not aligning well with the recording site. In a prior study, which investigated the signal correlation between EEG ear channels and EEG scalp channels [50], it was found that in-ear EEG signals have a higher correlation with signals from adjacent temporal lobe regions than other areas. Therefore, an in-ear EEG device should most efficiently receive signals originating from the temporal lobe.

The substantially lower intra-subject variation in alpha and beta band power observed in the mental rotation task compared to arithmetic and finger tapping likely explains the reduced classification accuracy. This phenomenon reflects the general stability of alpha and beta oscillatory dynamics during mental rotation. While effective workload classification relies on detecting distinct task-dependent shifts in oscillatory amplitude, the mental rotation task was characterized by remarkably steady alpha and beta oscillations across difficulty levels. This spectral stability meant that the oscillatory power changes failed to differentiate between difficulty levels, leaving the classification model insufficient discriminative information to reliably distinguish between different levels of mental workload.

An important observation is the substantial heterogeneity in within-subject stress regression performance across participants, with R^2^ values ranging from 0.41 to 0.99 (mean 0.76 ± 0.20). The exceptionally high performance for subject s80 warrants a careful examination. One plausible explanation relates to motion artifacts. The cold pressor task is inherently accompanied by increased motor activity (hand immersion, body tension responses), which typically generates muscle and motion artifacts in in-ear EEG recordings. Although our threshold-based artifact rejection approach effectively removes large-amplitude artifacts, residual low-amplitude muscle artifacts just below the rejection threshold could potentially become predictive features if they systematically co-occur with the cold pressor task. To determine whether s80’s result reflects superior signal quality versus overfitting to artifact-related characteristics, future work should employ cross-frequency analysis comparing high-frequency bands across subjects. Another phenomenon is that a minority of subjects (e.g., s87) reported limited variability in stress ratings, which may have affected the interpretation of R^2^ values for within-subject regression models. This observation may reflect individual differences in metacognitive awareness and self-assessment capabilities rather than a systematic failure of the EEG-based prediction model.

Although single-channel EEG devices offer portability and ease of use, their adoption in clinical and real-world settings faces significant limitations. Key challenges include reduced spatial resolution and susceptibility to artifacts [51]. The susceptibility to artifacts imposes certain constraints on the operational scope of portable EEG devices. For instance, the proposed system is not suitable for high-mobility scenarios, such as sports, where motion artifacts would heavily contaminate the signal. However, for the learning and professional working environments targeted in this study—where subjects are primarily stationary—the application proves highly feasible. Although sporadic motion artifacts are unavoidable even during sedentary behavior, the exclusion of contaminated data segments results in negligible data loss relative to the total recording duration. Consequently, this artifact rejection strategy does not compromise the system’s capacity to reliably monitor longitudinal trends in stress and mental workload. The most common single-channel EEG devices have electrodes placed on the forehead; this restricted spatial coverage makes it difficult to capture activities from parietal, temporal, and occipital regions, thus narrowing their application. The same applies to our system, where the electrodes are located near the temporal lobe; activities that are specific to other regions are hard to capture. Unlike multi-channel arrays that leverage spatial filtering to suppress noise, single-channel systems lack redundancy. Effective noise cancellation methods such as ICA-based noise removal are not applicable to single-channel signals. Other research also demonstrates that single-channel configurations miss critical neural events detectable in multi-channel setups, while algorithmic constraints limit their ability to generalize across diverse stressors [52]. Our work also demonstrated such limitations as the accuracy of classification varied greatly for different types of tasks. Furthermore, we acknowledge that there is still room for improvement in terms of model performance. To further enhance the accuracy and robustness of our stress and mental workload classification algorithms, future work should focus on expanding the scope of this research to include a larger and more diverse sample of subjects, as well as a broader range of learning tasks. By incorporating data from a wider variety of individuals and educational contexts, we can develop models that are more generalizable and better equipped to handle the complex, multifaceted nature of real-world learning.

## 5. Conclusions

In this study, we have successfully demonstrated the potential of a portable in-ear EEG device for real-time monitoring of stress and mental workloads in learning scenarios. By developing sophisticated machine learning models capable of extracting these critical cognitive states simultaneously, we have taken a significant step towards the realization of adaptive, EEG-based learning systems that can optimize educational outcomes by tailoring instructional interventions to individual learners’ needs. While the accuracy of our models may not yet rival that of some state-of-the-art approaches, it is important to consider the broader context in which these results were achieved. The in-ear EEG device employed in this study represents a major advance in terms of user-friendliness and comfort, making it an ideal candidate for everyday use in real-world learning settings. The unobtrusive nature of this device, combined with its ability to provide continuous, high-quality EEG data, opens up new avenues for the seamless integration of cognitive monitoring into educational practice.

## Figures and Tables

**Table 1:** Performance metrics of the cross-subject stress classification model on three-fold cross validation of eyes-open resting vs. cold pressor and meditation vs. cold pressor.

Tasks	Accuracy	Precision	Recall	F1 Score
Eyes open vs. cold pressor	0.78 ± 0.05	0.82 ± 0.01	0.72 ± 0.13	0.76 ± 0.07
Meditation vs. cold pressor	0.77 ± 0.14	0.81 ± 0.08	0.66 ± 0.26	0.71 ± 0.21

**Table 2:** Performance metrics of the within-subject stress regression model. Each row presents the metrics of the best fold for one subject.

Subject	$R^{2}$
s80	0.99
s82	0.48
s83	0.94
s84	0.78
s85	0.81
s87	0.41
s89	0.85
s92	0.83
Average	0.76
Standard deviation	0.21

**Table 3:** Performance metrics of the cross-subject mental workload binary classification model.

Task	Accuracy	Precision	Recall	F1
Arithmetic	0.79 ± 0.03	0.78 ± 0.08	0.85 ± 0.09	0.81 ± 0.00
Finger tapping	0.71 ± 0.10	0.68 ± 0.33	0.63 ± 0.23	0.57 ± 0.12
Mental rotation	0.59 ± 0.02	0.56 ± 0.05	0.90 ± 0.06	0.69 ± 0.02
Lexical decision	0.54 ± 0.06	0.53 ± 0.06	0.65 ± 0.11	0.58 ± 0.06
Arithmetic and finger tapping	0.74 ± 0.07	0.73 ± 0.14	0.72 ± 0.09	0.71 ± 0.03

## Data Availability

The data used in this study are available at https://cityucompuneurolab.github.io/data.html.

## Appendix A. EEG Signal Features

$$\begin{array}{cc} \mathrm{EEG}\;\mathrm{signal}:\; & y(t)\\ \mathrm{Mean}\;\mathrm{value}\;\mathrm{of}\;y(t):\; & \mu \\ \mathrm{Number}\;\mathrm{of}\;y(t)\;\mathrm{samples}:\; & N\\ \mathrm{Power}\;\mathrm{spectral}\;\mathrm{density},\;\mathrm{PSD}(f):\; & \lim_{T\to \infty }E\left[{\left|F\{y(t)\}(f)\right|}^{2}\right],\\ \; & \mathrm{where}\;T\;\mathrm{is}\;\mathrm{the}\;\mathrm{length}\;\mathrm{of}\;\mathrm{time}\;\mathrm{observed},\\ \; & F\{y(t)\}\;\mathrm{is}\;\mathrm{the}\;\mathrm{Fourier}\;\mathrm{transform}\;\mathrm{of}\;y(t),\\ \; & f\;\mathrm{is}\;\mathrm{the}\;\mathrm{frequency}.\\ \mathrm{Number}\;\mathrm{of}\;\mathrm{frequency}\;\mathrm{components}:\; & N_{f}\\ \mathrm{Frequency}\;\mathrm{resolution}:\; & ∆f \end{array}$$

**Hjorth features:** Activity, overall signal, activity(y(t))=var(y(t))
  Mobility, overall signal, $\mathrm{mobility}\left(y\left(t\right)\right)=\sqrt{\frac{var(\frac{dy(t)}{dt})}{var(y(t))}}$
  Complexity, overall signal, $\mathrm{complexity}\left(y\left(t\right)\right)=\frac{mobility(\frac{dy(t)}{dt})}{mobility(y(t))}$

**Statistical features:** Standard deviation, overall signal, $\mathrm{std}\left(y\left(t\right)\right)=\sqrt{\frac{{\sum |y\left(t\right)-\mu |}^{2}}{N}}$
  Sum of absolute derivative, overall signal, abs_diff(y(t))=∑|y(t+1)−y(t)|
  Skewness, overall signal, $\mathrm{skewness}\left(y\left(t\right)\right)=\frac{E[{\left(y\left(t\right)-\mu \right)}^{3}]}{\sigma^{3}}$
  Kurtosis, overall signal, $\mathrm{kurtosis}\left(y\left(t\right)\right)=\frac{E[{\left(y\left(t\right)-\mu \right)}^{4}]}{\sigma^{4}}$
  Mean, overall signal, $\mathrm{mean}\left(y\left(t\right)\right)=\frac{1}{N}\sum_{t=1}^{N}y\left(t\right)$
  Median, overall signal, median(y(t))
  Minimum value, overall signal, min(y(t))
  Maximum value, overall signal, max(y(t))
  Range, overall signal, range(y(t))

**Frequency domain features:** Relative power, delta band, $\mathrm{relative}\_\mathrm{delta}\left(y\left(t\right)\right)=\frac{\int_{0.1}^{4}PSD\left(f\right)df}{\int_{0.1}^{45}PSD\left(f\right)df}$
  Relative power, theta band, $\mathrm{relative}\_\mathrm{theta}\left(y\left(t\right)\right)=\frac{\int_{4}^{8}PSD\left(f\right)df}{\int_{0.1}^{45}PSD\left(f\right)df}$
  Relative power, alpha band, $\mathrm{relative}\_\mathrm{alpha}\left(y\left(t\right)\right)=\frac{\int_{8}^{12}PSD\left(f\right)df}{\int_{0.1}^{45}PSD\left(f\right)df}$
  Relative power, beta band, $\mathrm{relative}\_\mathrm{beta}\left(y\left(t\right)\right)=\frac{\int_{12}^{32}PSD\left(f\right)df}{\int_{0.1}^{45}PSD\left(f\right)df}$
  Relative power, gamma band, $\mathrm{relative}\_\mathrm{gamma}\left(y\left(t\right)\right)=\frac{\int_{32}^{45}PSD\left(f\right)df}{\int_{0.1}^{45}PSD\left(f\right)df}$
  Root-mean-square value, delta band, $\mathrm{rms}\_\mathrm{delta}\left(y\left(t\right)\right)=\sqrt{\frac{\sum |y_{\delta }\left(t\right)-\mu_{\delta }{|}^{2}}{N}}$
  Root-mean-square value, theta band, $\mathrm{rms}\_\mathrm{theta}\left(y\left(t\right)\right)=\sqrt{\frac{\sum |y_{\theta }\left(t\right)-\mu_{\theta }{|}^{2}}{N}}$
  Root-mean-square value, alpha band, $\mathrm{rms}\_\mathrm{alpha}\left(y\left(t\right)\right)=\sqrt{\frac{\sum |y_{\alpha }\left(t\right)-\mu_{\alpha }{|}^{2}}{N}}$
  Root-mean-square value, beta band, $\mathrm{rms}\_\mathrm{beta}\left(y\left(t\right)\right)=\sqrt{\frac{\sum |y_{\beta }\left(t\right)-\mu_{\beta }{|}^{2}}{N}}$
  Root-mean-square value, gamma band, $\mathrm{rms}\_\mathrm{gamma}\left(y\left(t\right)\right)=\sqrt{\frac{\sum |y_{\gamma }\left(t\right)-\mu_{\gamma }{|}^{2}}{N}}$
  Root-mean-square value, non-delta bands, $\mathrm{rms}\_\mathrm{non}\_\mathrm{delta}\left(y\left(t\right)\right)=\sqrt{\frac{\sum |\left[y\left(t\right)-y_{\delta }\left(t\right)\right]-\left[\mu -\mu_{\delta }\right]{|}^{2}}{N}}$
  Root-mean-square value, non-theta bands, $\mathrm{rms}\_\mathrm{non}\_\mathrm{theta}\left(y\left(t\right)\right)=\sqrt{\frac{\sum |\left[y\left(t\right)-y_{\theta }\left(t\right)\right]-\left[\mu -\mu_{\theta }\right]{|}^{2}}{N}}$
  Root-mean-square value, non-alpha bands, $\mathrm{rms}\_\mathrm{non}\_\mathrm{alpha}\left(y\left(t\right)\right)=\sqrt{\frac{\sum |\left[y\left(t\right)-y_{\alpha }\left(t\right)\right]-\left[\mu -\mu_{\alpha }\right]{|}^{2}}{N}}$
  Root-mean-square value, non-beta bands, $\mathrm{rms}\_\mathrm{non}\_\mathrm{beta}\left(y\left(t\right)\right)=\sqrt{\frac{\sum |\left[y\left(t\right)-y_{\beta }\left(t\right)\right]-\left[\mu -\mu_{\beta }\right]{|}^{2}}{N}}$
  Root-mean-square value, non-gamma bands, $\mathrm{rms}\_\mathrm{non}\_\mathrm{gamma}\left(y\left(t\right)\right)=\sqrt{\frac{\sum |\left[y\left(t\right)-y_{\gamma }\left(t\right)\right]-\left[\mu -\mu_{\gamma }\right]{|}^{2}}{N}}$
  Engagement formula, $\mathrm{engagement}\left(y\left(t\right)\right)=\frac{\mathrm{relative}\_\mathrm{beta}(y(t))}{\mathrm{relative}\_\mathrm{alpha}(y(t))+\mathrm{relative}\_\mathrm{theta}(y(t))}$
  Reciprocal of relative power, beta band, $1/\mathrm{beta}\left(y\left(t\right)\right)=\frac{1}{\mathrm{relative}\_\mathrm{beta}(y(t))}$
  Relative power ratio, beta to alpha bands, $\mathrm{beta}/\mathrm{alpha}\left(y\left(t\right)\right)=\frac{\mathrm{relative}\_\mathrm{beta}(y(t))}{\mathrm{relative}\_\mathrm{alpha}(y(t))}$
  Weighted average power, overall signal, $\mathrm{avg}\_\mathrm{power}\left(y\left(t\right)\right)=\frac{\sum_{i=1}^{N_{f}}PSD\left(i\cdot ∆f\right)\left(i\cdot ∆f\right)}{\sum_{i=1}^{N_{f}}PSD\left(i\cdot ∆f\right)}$
  Shannon entropy, overall signal, $\mathrm{PSD}\_\mathrm{norm}\left(y\left(t\right)\right)=\frac{PSD(y(t))-min[PSD(y(t))]}{max[PSD(y(t))]-min[PSD(y(t))]},\;\mathrm{entropy}\left(y\left(t\right)\right)=-\sum_{N_{f}}\mathrm{PSD}\_\mathrm{norm}\left(y\left(t\right)\right)\cdot log_{2}\left(\mathrm{PSD}\_\mathrm{norm}\left(y\left(t\right)\right)\right)$
  Power difference, beta band - alpha band, diff_ba(y(t))=relative_beta(y(t))−relative_alpha(y(t))
  Relative band power ratio, relative gamma / (relative theta + relative alpha), $\mathrm{RG}\left(y\left(t\right)\right)=\frac{\mathrm{relative}\_\mathrm{gamma}(y(t))}{\mathrm{relative}\_\mathrm{theta}(y(t))+\mathrm{relative}\_\mathrm{alpha}(y(t))}$
  Spectral edge frequency 90, $0.9\cdot \int_{-\infty }^{\infty }PSD\left(f\right)df=\int_{-\infty }^{\mathrm{sef}\_90(y(t))}PSD\left(f\right)df$
